# DNA barcoding of spiders from agricultural fields

**DOI:** 10.1080/23802359.2019.1693283

**Published:** 2019-11-20

**Authors:** Hafiz Muhammad Tahir, Muhammad Summer, Sana Mehmood, Sehrish Ashraf, Sajida Naseem

**Affiliations:** a Department of Zoology, Government College University Lahore, Lahore, Pakistan;; b Department of Zoology, University of Education Lower Mall Campus Lahore, Lahore, Pakistan

**Keywords:** Spiders, barcoding, agroecosystems, COI

## Abstract

In the present study, DNA barcoding was used to assess the percentage accuracy of morphological base identification of spiders from the agriculture fields of district Layyah, Punjab, Pakistan. A total of 872 spiders were captured from June to August of 2017. All the collected spiders were brought to molecular laboratory at GC University Lahore, preserved in 95% ethanol and stored at −20 °C until the DNA extraction. Spiders were evaluated morphologically on the basis of different identification Keys and Catalogs. Morphological identification revealed the presence of 12 families, 29 genra and 49 species. To evaluate the authenticity of morphological identification, tissue samples of 96 specimens were sent to Canadian Center for Biodiversity and Genomics, University of Guelph, Canada. A 658-base pair sequence of COI (Cytochrome c Oxidase Subunit I) of 90 specimens was retrieved successfully, which confirmed the presence of 11 families, 25 genra and 47 species. On the basis of molecular results, all the misidentified specimens were then allotted the correct taxon. Overall accuracy of morphological based identification was 88%. It is concluded from the present study that morphological investigations to identify a spider, are satisfactory but to enhance the accuracy, pace and credibility of results, molecular technique like DNA barcoding is considerable. Furthermore, to magnify authenticity of evaluation of spiders, integrated barcoding- combination of molecular methods and conventional taxonomy- is compulsory.

## Introduction

Spiders are the generalist predators and known as natural enemies of the pests (Maloney 2003). Their role in agricultural and forest ecosystems as a stabilizer of insect pest density, is very critical (Ghavami 2008).In contrast to specialist predators which preferred to feed on selective pest species, spiders have wide range of prey types (Sunderland and Samu 2000). Features like mortality of non-consumed pests in webs, excessive killing and partial consumption of prey, make spiders a strong bio-control agent.

Spider identification using morphological characteristics is time consuming and hectic job for different reasons (Barrett and Hebert 2005).Sexual dimorphism and absence of diagnostic characters in juveniles are the main hurdles in authentic evaluation of spiders (Robinson et al. 2009). Molecular identification techniques like DNA barcoding are being employed to overcome these kinds of problems (Bond et al. 2001). It is a novel technique used to deliver fast and cost-efficient species identification results with standard taxonomic information (Hebert and Gregory 2005; Miller 2007). This technique is based on the diversity of standardized regions (658 base pairs) of mitochondrial genome called biological barcode, which allows the species level identification (Hajibabaei et al. 2007). Organisms relating to different group including the bats (Clare et al. 2007), butterflies (Lukhtanov et al. 2009), birds (Kerr et al. 2007), fishes(Ward et al. 2005), diptera(Meier et al. 2006), algae (Saunders 2005), fungi (Seifert 2009; Schoch et al. 2012), amphibians (Vences et al. 2005), ants (Smith et al. 2005), crustaceans (Witt et al. 2006),wasps (Smith et al. 2008), and aphids have been successfully evaluated worldwide, using DNA barcoding.

DNA barcoding made great impacts not only in the successful molecular descriptions of already identified species but also assists in novel species discovery (Hebert et al. 2003; Hebert et al. 2004). The success and future of DNA Barcoding is dependent upon the assumption that genetic differences within a species are less than the differences between the species (Hebert et al. 2004; Hogg and Hebert 2004; Barrett and Hebert 2005; Smith et al. 2005; Ward et al. 2005; Hajibabaei et al. 2007). Scientists from all over the globe now gave a possible solution to the limitations of DNA barcoding and introduced the idea of “*integrated barcodes*” (Rubinoff 2006). Integrated barcoding involves the molecular and morphological approaches to identify and describe a species (Dayrat 2005; DeSalle et al. 2005; Gibbs 2009). The objective of study was to explore the undocumented spider’s fauna of the District Layyah, Punjab, Pakistan and efficacy of DNA barcoding in taxonomic evaluation. Establishment of genetic reference library for future study of spiders at molecular level was another motive of the study.

## Materials and methods

### Sampling of spiders

Spiders were collected from agricultural fields of District Layyah, Pakistan (Coordinates 29.436N, 68.877E and 30.966N, 70.950E). Some spiders were also collected from the leaf litter and demolished, old mud houses. Varieties of spiders were also sampled from Mango, Banana, *Acacia arabica* and Siris tree (*Albizia lebbeck*).

### Sampling methods

Different sampling methods including the pitfall, jerking, sweep net and hand picking were employed to catch the spiders (Robinson et al. 2009; Tahir et al. 2016). Pitfall method was used to collect the spiders from the ground surface. Foliage spiders were sampled through hand picking and sweep net while spiders from trees were captured by jerking (Robinson et al. 2009).

### Preservation technique

Collected spiders were transferred to the molecular laboratory of Zoology department, Government College University Lahore. All the specimens were then preserved in 96% ethanol. Until the DNA extraction, all the specimens were kept at −20 °C in the refrigerator.

### Morphological identification

Before applying the molecular technique for evaluation, spiders were identified on the basis of specific diagnostic morphological characters like body shape, eye pattern, epigyne structure and position of spinnerets. With the assistance of multiple available keys, morphological identification was made possible. Frequently used keys for identification were Barrian and Listinger (1995), Tikader and Malhotra (1980) and other available catalogs and literature.

### DNA extraction, PCR and gel electrophoresis

Tissue preparation for DNA extraction was done by cutting the leg of spider into small pieces with the aid of sharp sterilized blade onto a sterilized slide. These small fragments were the source for the extraction of genomic DNA with the help of Thermo Scientific GeneJet Genomic DNA purification kit. DNA was extracted by the column method of DNA extraction (GeneJET Genomic DNA Purification column) in which cell lysis was carried out by proteinase K, followed by incubation for overnight and resultant lysate was transferred to purification column. After that purification column containing lysate was centrifuged at 6000 rpm for 60 s and discarded the flow through. At last elution buffer was added to elute the DNA by centrifugation at 8000 × g.

Standard barcode (658 base pairs) region of mitochondrial COI gene was amplified through PCR at standard conditions. The forward and reverse primers which were used for DNA amplification were as follows

- C_LepFolF/C_LepFolR

For verification of the PCR product, Agarose 1% was used for gel electrophoresis and resultant bands were analyzed by comparing with the ladder (Fermentas #1173) of known size. Ethidium bromide was added to visualize the bands under UV illuminator.

### DNA sequencing

DNA sequencing was performed in collaboration with Center for Biodiversity and Genomics, University of Guelph, Canada. Generated sequences were submitted to BOLD in our already developed project MTSPD. Then MEGA 5.2 software was consulted to align the present study sequences. To compute the barcode gap which arises when the interspecific genetic divergences exceeds the intra-specific divergences, we used the BOLD online system v3. By applying the Kimura 2 parameter as a distance model, COI-5P- Cytochrome Oxidase Subunit 1 5′ Region as a marker, BOLD Aligner as a sequence aligner and sequence length of ≥600 base pairs as a filter in BOLD software, we generated the barcode gaps of all the under study specimens.

## Results

### Morphology-based identification

**Figure 1.**
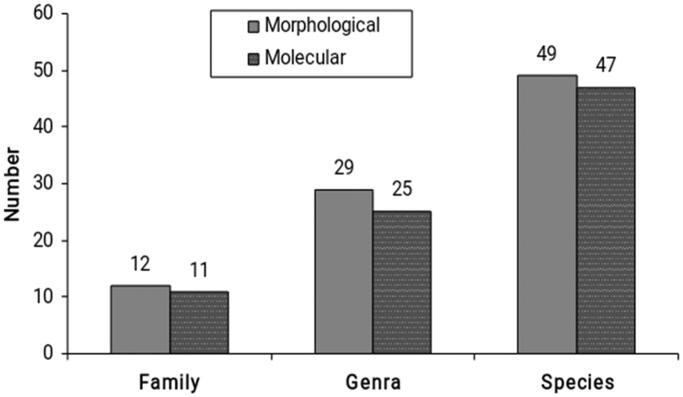
Comparison of morphological vs molecular evaluation of spiders.

A total of a total of 872 specimens representing 12 families, 29 genra and 49 species were identified on morphological basis (Figure 1). Out of the total catch, 759 specimens were adult and remaining 113 immature. List of morphologically identified species is given in the Table 1. The most abundant family on the ground was Salticidae. However, family Oxyopidae was most common on foliage. Family Lycosidae was mostly found under fallen leaves, detritus material and soil crevices in the fields. Family Araneidae was represented by highest number of individuals followed by Clubionidae, Gnaphosidae, Oecobiidae, Sparassidae, tetragnathidae, Thomisidae, Eutichuridae, Scytodidae and Hahnidae.

**Table 1.** Details of morphological-based identified and described spiders in the present study.

Family name	Species name	Specimen number	Relative abundance (%)
Arenidae	*Eriovixia excelsa (*Simon 1889)	237	27.17
	*Neoscona theisi* (Walckenaer 1841)		
	*Neoscona rumpfi* (Thorell 1878)		
	*Neoscona usbonga* (Barrion and Listinger 1995)		
	*Larinia phthisica* (Simon 1874)		
	*Argiope fasciata* (Forskål 1775)		
Eutichuridae	*Cheiracanthium inclusum* (Hentz 1847)	42	4.81
	*Cheiracanthium sp.*		
Gnaphosidae	*Gnaphosa jodhpurensis* (Tikader and Gajbe 1977)	35	4.01
	*Gnaphosidae sp.*		
	*Gnphosidae sp.2*		
	*Gnphosidae sp.3*		
	*Gnphosidae sp.4*		
Hahnidae	*Isopeda sp.*	1	0.114
Lycosidae	*Arctosa sp.*	158	18.11
	*Pardosa apostolic*		
	*Hippasa partita* (O.P. Cambridge 1876)		
	*Venonia gabrielae*		
	*Trochosa terricola* (Thorell 1856)		
	*Pardosa distincta* (Blackwall 1846)		
	*Trochosa alveoli*		
	*Pirata sp.*		
	*Pardosa birmanica* (Simon 1844)		
	*Lycosidae sp.*		
	*Arctosa tanakai* (Barrion and Litsinger 1995)		
	*Hippasa holomerae* (Thorell 1895)		
	*Pardosa sumatrana* (Thorell 1890)		
Oecobidae	*Oecobius sp.*	33	3.78
	*Oecobius sp1*		
	*Oecobius sp3*		
	*Oecobius sp4*		
Oxypidae	*Oxyopes tiengianensis* (Barrion and Litsinger 1995)	131	15.02
	*Oxyopes matiensis* (Barrion and Litsinger 1995)		
	*Oxyopes aspirasi* (Barrion and Litsinger 1995)		
	*Oxopes sp 4*		
	*Peuccetia sp.*		
	*Oxyopes pingasus* (Barrion and Litsinger 1995)		
Salticidae	*Pseudicius admirandus* (Logunov 2007)	173	19.83
	*Plexippus paykulli* (Audouin 1826)		
	*Phntella vittata* (Koch 1846)		
	*Thyene imperialis* (Rossi 1846)		
	*Phlegra fasciata* (Hahn 1826)		
Scytodidae	*Scytodes thoracica* (Latreille 1802)	11	1.26
Sparassidae	*Olios mahabangkawitus* (Barrion and Litsinger 1995)	16	1.83
Tetragnathidae	*Leucauge decorata* (Walckenaer 1841)	13	1.49
Thomisidae	*Thomisus okinawensis* (Strand 1907)	23	2.6
	*Runcinia albostriata* (Boesenberg and Strand 1906)		
**Total**		**872**	**100**

### Genetic-based identification

**Figure 2.**
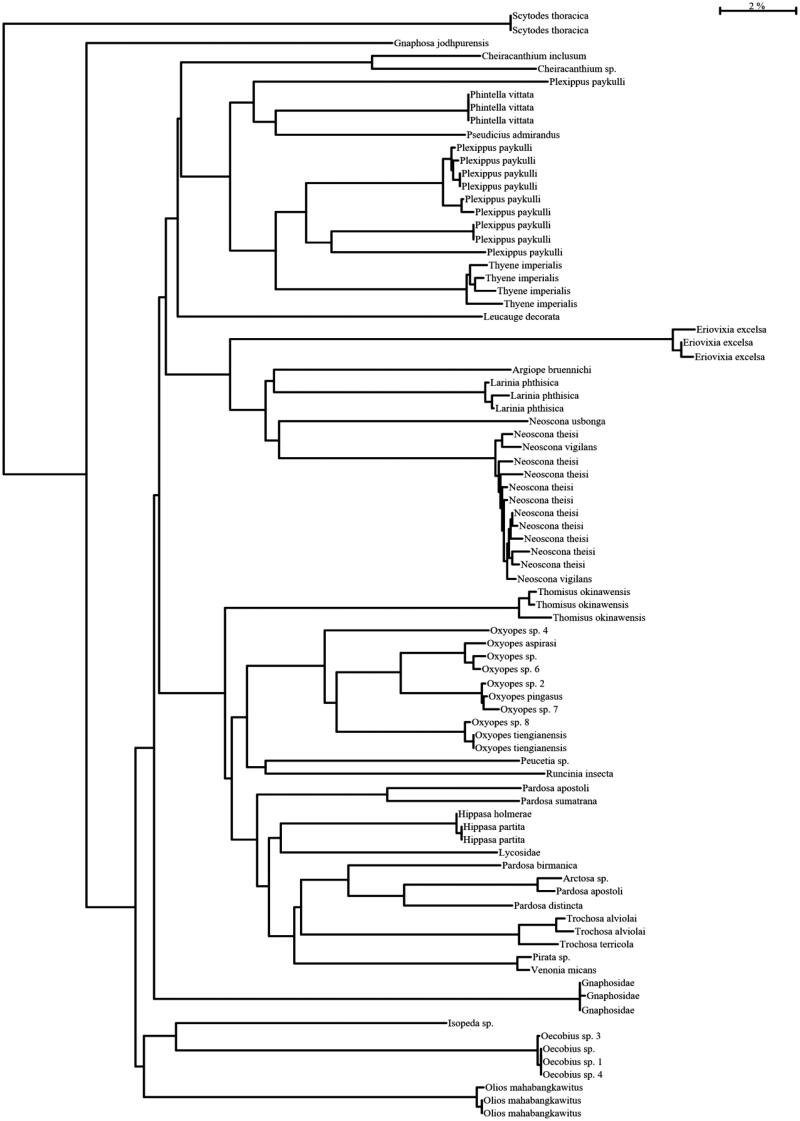
Neighbor joining tree of spiders collected from agricultural fields.

To verify the authenticity of morphology-based identification of spiders, 95 specimens were subjected to DNA barcoding. Genomic sequence of COI up to 600 base pairs was successfully retrieved from 90 individuals. Morphological identification failed in precise evaluation of 11 specimens, which were then allotted the correct taxon on the premise of biological barcode sequence as depicted in the Table 2. DNA barcoding affirm the presence of 11 families, 25 genra and 47 species as shown in Figure 2. Overall, accuracy of morphology-based identification was 88%. Figure 2 demonstrates the neighbor joining tree of all the specimens.

**Table 2.** Morphologically misidentified specimens alongwith their correct taxon.

Morphological identification	Molecular identification
*Cheiracanthium sp*	*Cheiracanthium inclusum*
*Neoscona rumfi*	*Neoscona vigilans*
*Neoscona rumfi*	*Neoscona vigilans*
*Oxyopes sp*	*Oxyopes aspirasi*
*Oxyopes sp 6*	*Oxyopes aspirasi*
*Oxyopes sp 2*	*Oxyopes pingasus*
*Oecobius sp*	*Oecobius sp 3*
*Runcinia albostriata*	*Runcinia insect*
*Hippasa partita*	*Hippasa holomerae*
*Isopeda sp.*	*Oecobius sp.*
*Venonia gabrielae*	*Venoniia micans*

**Table 3.** Specimens BOLD ID’s with their taxonomic identification and coordinates of the collection sites are given below.

	BOLD ID	Taxonomic identification	Coordinates of collection sites
1	GCUL-MTSPD-476	*Pseudicius admirandus*	30.966413, 70.950973
2	GCUL-MTSPD-477	*Plexippus paykulli*	30.966413, 70.950973
3	GCUL-MTSPD-478	*Phntella vittata*	30.966413, 70.950973
4	GCUL-MTSPD-479	*Thyene imperialis*	30.966413, 70.950973
5	GCUL-MTSPD-480	*Plexippus paykulli*	30.966413, 70.950973
6	GCUL-MTSPD-481	*Thyene imperialis*	30.966413, 70.950973
7	GCUL-MTSPD-482	*Plexippus paykulli*	30.966413, 70.950973
8	GCUL-MTSPD-483	*Plexippus paykulli*	30.966413, 70.950973
9	GCUL-MTSPD-484	*Thyene imperialis*	30.966413, 70.950973
10	GCUL-MTSPD-485	*Phintella vittata*	30.966413, 70.950973
11	GCUL-MTSPD-486	*Phintella vittata*	30.966413, 70.950973
12	GCUL-MTSPD-487	*Plexippus paykulli*	30.966413, 70.950973
13	GCUL-MTSPD-488	*Phlegra fasciata*	30.966413, 70.950973
14	GCUL-MTSPD-489	*Plexippus paykulli*	30.966413, 70.950973
15	GCUL-MTSPD-490	*Thyene imperialis*	30.966413, 70.950973
16	GCUL-MTSPD-491	*Plexippus paykulli*	30.966413, 70.950973
17	GCUL-MTSPD-492	*Plexippus paykulli*	30.966413, 70.950973
18	GCUL-MTSPD-493	*Plexippus paykulli*	30.966413, 70.950973
19	GCUL-MTSPD-494	*Plexippus paykulli*	30.966413, 70.950973
20	GCUL-MTSPD-495	*Plexippus paykulli*	30.966413, 70.950973
21	GCUL-MTSPD-496	*Thomisus okinawensis*	30.966413, 70.950973
22	GCUL-MTSPD-497	*Thomisus okinawensis*	30.966413, 70.950973
23	GCUL-MTSPD-498	*Thomisus okinawensis*	30.966413, 70.950973
24	GCUL-MTSPD-499	*Runcinia insect*	30.966413, 70.950973
25	GCUL-MTSPD-500	*Arctosa sp.*	30.966413, 70.950973
26	GCUL-MTSPD-501	*Pardosa apostolic*	30.966413, 70.950973
27	GCUL-MTSPD-502	*Hippasa partita*	30.966413, 70.950973
28	GCUL-MTSPD-503	*Venonia gabrielae*	30.966413, 70.950973
29	GCUL-MTSPD-504	*Trochosa terricola*	30.966413, 70.950973
30	GCUL-MTSPD-505	*Pardosa distincta*	30.966413, 70.950973
31	GCUL-MTSPD-506	*Hippasa partita*	30.966413, 70.950973
32	GCUL-MTSPD-507	*Trochosa alveoli*	30.966413, 70.950973
33	GCUL-MTSPD-508	*Pirata sp.*	30.966413, 70.950973
34	GCUL-MTSPD-509	*Trochsa alveoli*	30.966413, 70.950973
35	GCUL-MTSPD-510	*Pardosa apostolic*	30.966413, 70.950973
36	GCUL-MTSPD-511	*Pardosa birmanica*	30.966413, 70.950973
37	GCUL-MTSPD-512	*lycosidae sp.*	30.966413, 70.950973
38	GCUL-MTSPD-513	*Arctosa tanakai*	29.435622, 68.876546
39	GCUL-MTSPD-514	*Hippasa holomerae*	29.435622, 68.876546
40	GCUL-MTSPD-515	*Pardosa sumatrana*	29.435622, 68.876546
41	GCUL-MTSPD-516	*Eriovixia excelsa*	29.435622, 68.876546
42	GCUL-MTSPD-517	*Neoscona theisi*	29.435622, 68.876546
43	GCUL-MTSPD-518	*Neoscona theisi*	29.435622, 68.876546
44	GCUL-MTSPD-519	*Neoscona rumfi*	29.435622, 68.876546
45	GCUL-MTSPD-520	*Neoscona theisi*	29.435622, 68.876546
46	GCUL-MTSPD-521	*Neoscona usbonga*	29.435622, 68.876546
47	GCUL-MTSPD-522	*Neoscona theisi.*	29.435622, 68.876546
48	GCUL-MTSPD-523	*Neoscona theisi*	29.435622, 68.876546
49	GCUL-MTSPD-524	*Eriovixia excelsa*	29.435622, 68.876546
50	GCUL-MTSPD-525	*Neoscona theisi.*	29.435622, 68.876546
51	GCUL-MTSPD-526	*Eriovixia excelsa*	29.435622, 68.876546
52	GCUL-MTSPD-527	*Neoscona rumfi*	29.435622, 68.876546
53	GCUL-MTSPD-528	*Neoscona theisi*	29.435622, 68.876546
54	GCUL-MTSPD-529	*Neoscona theisi*	29.435622, 68.876546
55	GCUL-MTSPD-530	*Neoscona theisi*	29.435622, 68.876546
56	GCUL-MTSPD-531	*Neoscona theisi*	29.435622, 68.876546
57	GCUL-MTSPD-532	*Oxyopes tiengianensis*	29.435622, 68.876546
58	GCUL-MTSPD-533	*Oxyopes matiensis*	29.435622, 68.876546
59	GCUL-MTSPD-534	*Oxyopes aspirasi*	29.435622, 68.876546
60	GCUL-MTSPD-535	*Oxyopes aspirasi*	29.435622, 68.876546
61	GCUL-MTSPD-536	*Peucetia sp.*	29.435622, 68.876546
62	GCUL-MTSPD-537	*Oxyopes pingasus*	30.966413, 70.950973
63	GCUL-MTSPD-538	*Oxyopes sp 3*	30.966413, 70.950973
64	GCUL-MTSPD-539	*Oxyopes sp*	30.966413, 70.950973
65	GCUL-MTSPD-540	*Oxyopes sp 5*	30.966413, 70.950973
66	GCUL-MTSPD-541	*Oxyopes aspirasi*	30.966413, 70.950973
67	GCUL-MTSPD-542	*Oxyopes tiengianensis*	30.966413, 70.950973
68	GCUL-MTSPD-543	*Oxyopes pingasus*	30.966413, 70.950973
69	GCUL-MTSPD-544	*Oxyopes pingasus*	30.966413, 70.950973
70	GCUL-MTSPD-545	*Oxyopes tiengianensis*	30.966413, 70.950973
71	GCUL-MTSPD-546	*Leucauge decorate*	30.966413, 70.950973
72	GCUL-MTSPD-547	*Larinia phthisica*	30.966413, 70.950973
73	GCUL-MTSPD-548	*Larinia phthisica*	30.966413, 70.950973
74	GCUL-MTSPD-549	*Larinia phthisica*	30.966413, 70.950973
75	GCUL-MTSPD-550	*Larinia phthisica*	30.966413, 70.950973
76	GCUL-MTSPD-551	*Larinia phthisica*	30.966413, 70.950973
77	GCUL-MTSPD-552	*Argiope fasciata*	30.966413, 70.950973
78	GCUL-MTSPD-553	*Gnaphosa jodhpurensis*	30.966413, 70.950973
79	GCUL-MTSPD-554	*gnaphosidae sp*	30.966413, 70.950973
80	GCUL-MTSPD-555	*gnaphosidae sp 2*	30.966413, 70.950973
81	GCUL-MTSPD-556	*gnaphosidae sp 3*	30.966413, 70.950973
82	GCUL-MTSPD-557	*gnaphosidae sp 4*	30.966413, 70.950973
83	GCUL-MTSPD-558	*Scytodes thoracica*	30.966413, 70.950973
84	GCUL-MTSPD-559	*Scytodes thoracica*	30.966413, 70.950973
85	GCUL-MTSPD-560	*Olios mahabangkawitus*	30.966413, 70.950973
86	GCUL-MTSPD-561	*Olios mahabangkawitus*	30.966413, 70.950973
87	GCUL-MTSPD-562	*Olios mahabangkawitus*	30.966413, 70.950973
88	GCUL-MTSPD-563	*Olios mahabangkawitus*	30.966413, 70.950973
89	GCUL-MTSPD-564	*Cheiracanthium inclusum*	30.966413, 70.950973
90	GCUL-MTSPD-565	*Cheiracanthium sp*	30.966413, 70.950973
91	GCUL-MTSPD-566	*Isopeda sp.*	30.966413, 70.950973
92	GCUL-MTSPD-567	*Oecobius sp.*	30.966413, 70.950973
93	GCUL-MTSPD-568	*Oecobius sp 1*	30.966413, 70.950973
94	GCUL-MTSPD-569	*Oecobius sp 3*	30.966413, 70.950973
95	GCUL-MTSPD-570	*Oecobius sp 4*	30.966413, 70.950973

## Discussion

The main goal of our study was to explore the spider’s diversity of district Layyah and to compare the efficacy of DNA barcoding with morphological-based evaluation for species identification. Investigation of the suitability of DNA barcoding for examining the genetic variations among the 29 genra was another motive behind the present study. Comparing to molecular-based evaluation, morphological-based identification success rate was 88%. Absence of diagnostic characters and availability of identification keys for juvenile spiders could be the possible factors for low success rate. DNA sequence retrieval of 5 specimens belonging to different genra was failed. Degradation of DNA due to inappropriate preservation techniques and primers mismatch could be the possible explanation behind this failure. Dean and Ballard (2001) and Kress and Erickson (2008) also described that improper preservation methods and primer mismatch could damage the DNA permanently.

In the present study, a total of 49 spiders species were identified morphologically belonging to the family Araneidae, Lycosidae, Oxyopidae, Salticidae, Sparassidae, Scytodidae, Gnaphosidae, Hahnidae, Oecobiidae, Tetragnathidae and Thomisidae. We could report only a proportion of spider’s species due to insufficient sampling effort. Although, many researchers across the Pakistan have reported large number of species. Overall, in 872 specimens, Family Araneidae catch was the highest. Sharma et al. (2010) described Araneidae as the most abundant family followed by the Salticidae in rice field. Family Lycosidae was dominant on ground which is reported by many researchers. Tahir et al. (2015) reported Lycosidae as a dominant family on ground. During the present study, hand picking and jerking method were used for spider’s collection. Robinson et al. (2009) also used the hand picking and jerking methods for sampling.

The 5′ end of Biological barcode (COI) was selected for species discrimination through DNA barcoding because of availability of the primers for recovery of required DNA from wide range of taxa (Hebert et al. 2003). In morphological evaluation, we misidentified 11 specimens and then according to the molecular results, we allotted them their correct taxa. This evaluation of specimens justified the identification power of DNA barcoding for specimens with fewer diagnostic characters. Goldstein and DeSalle (2003) reported the recovery of DNA from century old specimens thorough molecular techniques. We concluded from these results that molecular methods like DNA barcoding are necessary for complete and accurate species identification. Hebert et al. (2003, 2004) reported the DNA barcoding, a technique with 100% accuracy. Neighbor joining tree separated the specimens into different species with genetic difference of 2% or more in the present study. For family Araneidae, a significant barcode gap was also observed between the intra and inter-specific divergences. Furthermore, maximum intra specific values were less than the distance to NN. Slowik and Blagoev (2012) reported the same results for family Araneidae as we did in our study. There was no overlap between the intra and inter-specific values for family Araneidae in present study but Čandek and Kunter (2015) found the overlapping in the divergences values for family Araneidae.

For family Eutichuridae, Gnaphosidae, Scytodidae, Sparassidae and Tetragnathidae it was impossible to calculate the inter and intra-specific divergences as well as distances to NN because of the small sample size of these families. No overlap was found between the inter and intra-specific values of family Lycosidae. These values are in accordance with the results of Čandek and Kunter (2015), who found 100% accuracy result in evaluating the specimens of family Lycosidae. Robinson et al. (2009) described that the maximum intra-specific divergences are less than the distance to NN in the family Lycosidae. However, in the present study, *Pardosa birmanica* showed more than 98% resemblance with sequences of *Wedicosa fidelis,* when matched with the available sequences at the Genebank. Many researchers like Naseem and Tahir (2018) across the Pakistan reported this species as *Pardosa birmanica.* There is still confusion in the exact taxon of this specimen which is needed to be rectify.

Great ambiguity was recorded during the morphological identification of family Oxyopidae specimens due to variations of color and body patterns. Three specimens of the *Oxyopes matiensis* were misidentified as a different species due to differences in these patterns. Bond et al. (2001) also reported the problem of morphological differences in the same species and suggested the molecular approaches like DNA barcoding to overcome these hurdles. After the results of DNA barcoding, those misidentified specimens were allotted their exact taxon. Robinson et al. (2009) described hybridization, introgression and quick morphological divergences as the possible causes for these kinds of variations. Approach of “integrated barcoding” was also used by Slowik and Blagoev (2012) to overcome these types of issues. A clear barcode gap was analyzed for the family Salticidae. There was no overlap between the intra and inter-specific divergences. However, Čandek and Kunter (2015) reported an overlap of the intra and inter-specific values.

For the total of 90 specimens of present study, a significant barcode gap was observed in the intra and inter-specific divergences indicating the reliability of the results (Naseem and Tahir 2018). Moreover, values of maximum intra-specific divergences were lower than the distance to NN for every species. These results showed the 100% accuracy in identifying the juvenile and adult spiders using the molecular method in this study. These 100% successful results are in accordance with the results of Barrett and Hebert (2005), who correctly evaluated the 168 species of spiders using molecular tool of DNA barcoding. Čandek and Kunter also suggested the use of DNA barcoding for evaluation of spider’s species. Tahir et al. (2016) also identified 5 spider species with 100% success using DNA barcoding. Robinson et al. (2009) also successfully described the 19 species-rich genra using DNA barcoding. All these results validate the point of relying on DNA barcoding for highly accurate and authentic results for species evaluation.

DNA barcoding has appeared to be a standard species discriminatory technique due to its cheap, fast and authentic results (Tahir et al. 2016). In conclusion, we can say that morphological based approaches to describe any spider species are satisfactory but to magnify the pace and credibility of the results, combination of DNA barcoding is advantageous.
